# Comparison of SSS-CN and PHQ-15 in the evaluation of patients with suspected psychological disorders in cardiovascular medicine

**DOI:** 10.3389/fpsyg.2023.1027253

**Published:** 2023-03-02

**Authors:** Yan Fu, Qiuzhen Lin, Qunyan Xiang, Xingyu Wen, Ling Liu

**Affiliations:** ^1^Department of Cardiovascular Medicine, The Second Xiangya Hospital, Central South University, Changsha, Hunan, China; ^2^Research Institute of Blood Lipid and Atherosclerosis, Central South University, Changsha, Hunan, China; ^3^Modern Cardiovascular Disease Clinical Technology Research Center of Hunan Province, The Second Xiangya Hospital, Central South University Changsha, Hunan, China; ^4^Cardiovascular Disease Research Center of Hunan Province, The Second Xiangya Hospital, Central South University, Changsha, Hunan, China; ^5^Xiangya School of Medicine, Central South University, Changsha, Hunan, China

**Keywords:** SSS-CN, PHQ-15, somatic symptom disorder, anxiety, depression, screen

## Abstract

**Aims:**

Somatic Symptom Scale-China (SSS-CN) has been applied to assess the presence and severity of somatization symptom disorders (SSD) in Chinese patients. However, there was no study comparing SSS-CN with Patient Health Questionnaire-15 (PHQ-15). The aim of this study was to compare the consistency of the SSS-CN with the PHQ-15 in evaluating SSD in patients with suspected psychological disorders in cardiovascular medicine and to explore the relationship between scores on the two SSD self-rating scales and scores on self-rating scales for anxiety or depression.

**Methods:**

In this study, 1,324 subjects were enrolled by using a “three-question method.” Then, they completed four self-assessment scales, i.e., SSS-CN, PHQ-15, Patient Health Questionnaire-9 (PHQ-9), and General Anxiety Disorder-7 (GAD-7), in turn. The ability of SSS-CN to diagnose SSD was analyzed by the receiver operating characteristic (ROC) curve, and the area under the curve (AUC) value, sensitivity, and specificity were calculated. Reliability analysis was performed with the *Kappa* statistic to determine consistency between SSS-CN and PHQ-15. The relationship between two qualitative variables was analyzed by *Spearman* correlation analysis.

**Results:**

The proportions of SSD evaluated by SSS-CN and PHQ-15 were 83.2 and 87.0%, respectively. SSS-CN score was significantly correlated with PHQ-15 one (*r* = 0.709, *p* < 0.001). The AUC of the SSS-CN for the diagnosis of SSD was 0.891, with a high sensitivity and acceptable specificity. There was a moderate agreement between SSS-CN and PHQ-15 in assessing SSD, with a *Kappa* value of 0.512. Anxiety and/or depression were detected in about 70% of patients with SSD. There was significant correlation between the score of each SSD scale and that of GAD-7 or PHQ-9 (SSS-CN: *r* = 0.614 or 0.674; PHQ-15: *r* = 0.444 or 0.582, all *p* < 0.001). In addition, the SSS-CN score was more closely correlated with the GAD-7 or PHQ-9 score than the PHQ-15 score, and a higher proportion of patients with anxiety or depression was detected in those with moderate and severe SSD evaluated by SSS-CN.

**Conclusion:**

The SSS-CN could be one of the ideal scales for the rapid screening of patients with suspected psychological disorders in cardiovascular medicine.

## 1. Introduction

Currently, psychological disorders show increasing prevalence worldwide. Moreover, according to a multicenter cross-sectional survey in the United States, in over 50% of cases, comorbidities existed between depression, anxiety and somatization (Löwe et al., 2008). Commonly, cardiovascular diseases are accompanied by psychological disorders in clinical practice. This phenomenon has been found not only in Japanese hospitals but also in European ones (Suzuki et al., 2011; Celano et al., 2016; Matsuda et al., 2017; Aguirre-Camacho and Moreno-Jiménez, 2018; Norlund et al., 2018; Reavell et al., 2018). Psychological disorders have various manifestations, including chronic generalized anxiety, depression, the coexistence of depressive and anxiety symptoms, somatization symptom disorders (SSD), etc. (de Waal et al., 2004; Kroenke et al., 2007; Kirkcaldy and Siefen, 2012). Among them, SSD is one of the most common categories in the outpatient clinics of general hospitals (Hatcher and Arroll, 2008; Kurlansik and Maffei, 2016). It was estimated that the prevalence of SSD in the general population could be 5 to 7% (American Psychiatric Association, 2013). Recent evidence showed that the prevalence of SSD in cardiology outpatients was 64.2% in China (Ye et al., 2013). Moreover, SSD is usually accompanied by other types of psychological disorders, such as anxiety and/or depression. Therefore, early identification of SSD by clinicians is of great importance.

However, the recognition rate of psychological disorders by non-psychiatric clinicians was relatively low in China (He et al., 2010; Zhuang et al., 2010; Lyu et al., 2019; Zhang et al., 2019). The rate of recognition of depressive disorders in internal medicine outpatient departments of 23 general hospitals in Shenyang was observed only 4.0% in a Chinese study (Qin et al., 2008). Whereas another study indicated that the rate of SSD was 93.1% in general hospitals, such as in the department of cardiology (Zhang et al., 2019). It may lead to repeated visits by the patients, excessive examinations, and inaccurate treatment, finally resulting in wasted medical resources, increasing the financial burden on the patients, and even exacerbating doctor-patient conflicts. Thus, effective tools are necessary to help non-psychiatric clinicians to screen SSD in suspected patients.

Currently, Patient Health Questionnaire-15 (PHQ-15) is the most widely used scale to screen SSD. It has well-established psychometric properties with multiple language versions and has been used in large-scale studies (Zhu et al., 2012; Hyphantis et al., 2014; Hinz et al., 2017; Leonhart et al., 2018; Ran et al., 2020; Toussaint et al., 2020; Stauder et al., 2021). Somatic Symptom Scale-China (SSS-CN) is a scale inquiring about somatic and psychological symptoms developed by Zhuang et al. (2010) in 2010, and is derived from the DSM-5 and designed to assess the presence and severity of SSD. It has been verified to present high reliability and validity in many Chinese studies with a small sample size (Li et al., 2017; Chen et al., 2020; Tian et al., 2021). To date, few studies have compared SSS-CN and PHQ-15, and there was only one study protocol recently raised by Jiang et al. (2019). The aim of this study was to compare the consistency of the SSS-CN with the PHQ-15 in evaluating SSD in patients with suspected psychological disorders in cardiovascular medicine and to explore the relationship between scores on the two SSD self-rating scales and scores on self-rating scales for anxiety or depression.

## 2. Methods

### 2.1. Procedure and participants

In this study, 3,000 patients with suspected psychological disorders were recruited from July 2018 to January 2020 in the Department of Cardiovascular Medicine, Second Xiangya Hospital, Central South University. All of them had been screened by a “three-questions method” recommended by the Chinese expert consensus on psychological prescription for the patients in the department of cardiovascular medicine in 2020 (Committee of Cardiac Rehabilitation and Prevention of Chinese Association of Rehabilitation Medicine et al., 2020). The three questions were as follows: (1) do you have trouble sleeping? (2) do you feel anxious? and (3) do you have any physical discomfort that cannot be explained by multiple examinations? If the answer to at least two of the three questions was “yes,” there was an approximately 80% probability of having a psychological disorder (Committee of Cardiac Rehabilitation and Prevention of Chinese Association of Rehabilitation Medicine et al., 2020), i.e., a patient with a suspected psychological disorder no matter he/she had a cardiovascular disease or not. Finally, 2,500 patients were screened in this study.

**Figure 1:**
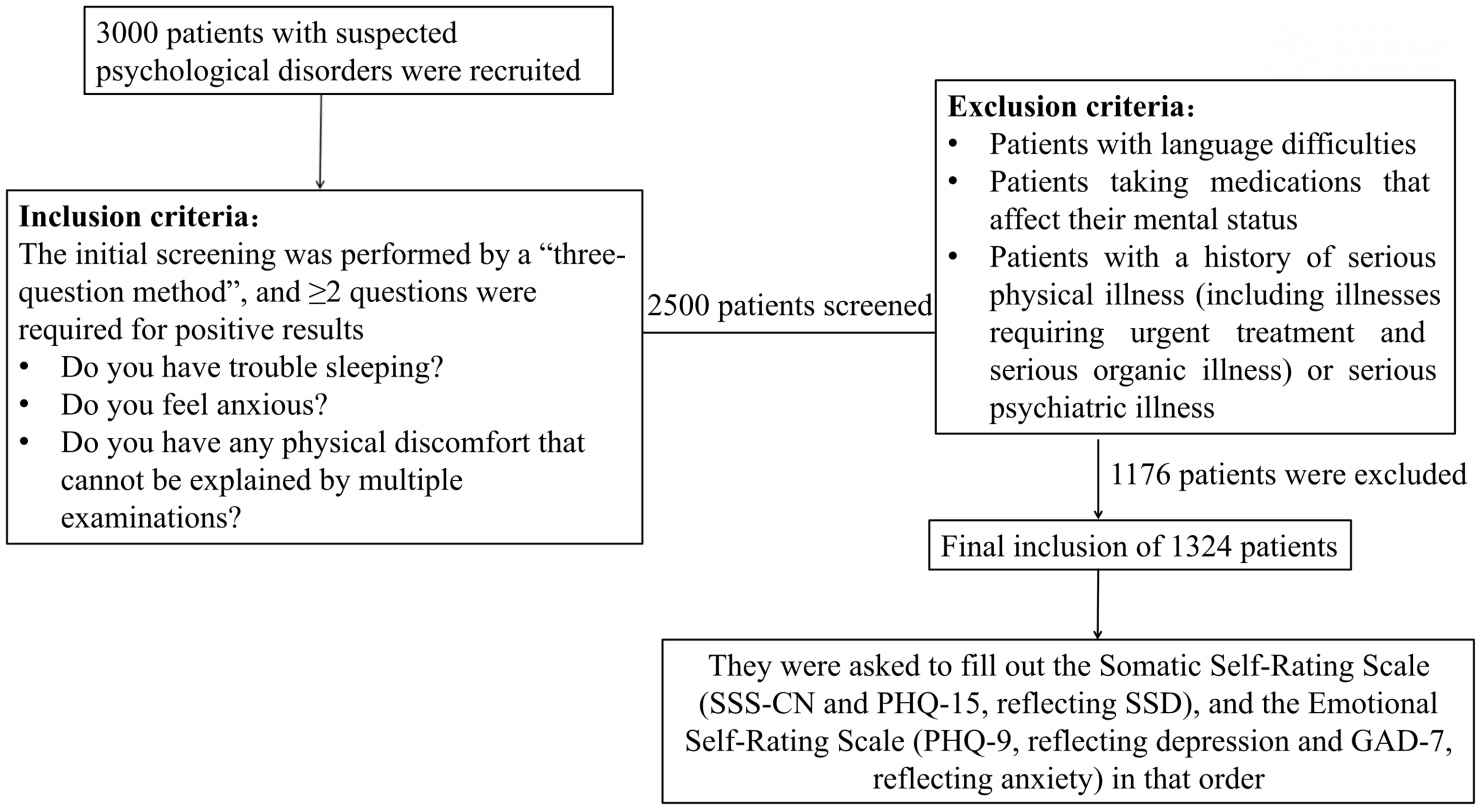
Flowchart of research subject enrollment.

The exclusion criteria were as follows: patients with language difficulties, those taking drugs that can affect their mental states, those with a history of severe physical illnesses (including illnesses that require emergency treatment and severe organic diseases), or severe mental illnesses. 1,176 patients met the exclusion criteria. A final total of 1,324 patients were included. They were 18–79 years old and consisted of 777 (58.7%) women and 547 (41.3%) men. Then, all suspected patients completed four self-assessment scales, including SSS-CN, PHQ-15, Patient Health Questionnaire-9 (PHQ-9), and General Anxiety Disorder-7 (GAD-7), to assess their somatic and psychological status (see flow chart Figure 1).

### 2.2. Assessment instruments

#### 2.2.1. SSS-CN

SSS-CN is a 20-item self-reported scale that assesses somatic symptom features. It contains 10 items assessing somatic symptoms (items 1, 5, 9, 10, 12, 13, 16, and 18–20), 4 items assessing depression (items 3, 4, 7, and 11), 4 items assessing anxiety (items 6, 14, 15, and 17) and 2 items assessing both anxiety and depression (items 2 and 8), which was described in detail by Jiang et al. (2019). The response options for each symptom are scored from 1 (‘does not exist’) to 4 (‘the problem occurred almost every day and/or is unendurable’) and the total score ranges from 20 to 80. The sum of the scores determines the presence and severity of SSD. The total scores in the ranges 20–29, 30–39, 40–59, and ≥ 60 were defined as “normal,” “mild SSD,” “moderate SSD” and “severe SSD,” respectively. One was taken as negative while ≥2 was regarded as positive for each item of SSS-CN. A total score ≥ 30 was considered positive for SSS-CN evaluation.

#### 2.2.2. PHQ-15

PHQ-15 is a 15-item self-report health questionnaire developed by Kroenke et al. (2002) and used to assess the number and severity of physical symptoms. Patients were required to respond to how much they had been troubled by 15 somatic symptoms or symptom clusters within the last 4 weeks. Each symptom can be scored from 0 (not bothered at all) to 2 (bothered a lot). Sum scores range from 0 to 30. Higher scores indicate a higher self-rated symptom burden. The total scores in the ranges 0–4, 5–9, 10–14, and, 15–30 were defined as “normal,” “mild SSD,” “moderate SSD” and “severe SSD,” respectively. Zero was taken as negative while ≥1 was regarded as positive for each item of PHQ-15. A total score ≥ 5 was considered positive for the PHQ-15 evaluation.

#### 2.2.3. GAD-7

GAD-7 is a 7-item, self-rated scale developed by Spitzer et al. (2006) to assess the degree of generalized anxiety disorder as well as other common anxiety disorders among patients in non-psychiatric hospital clinics in the last 2 weeks. Each item of this scale ranges from 0 to 3, representing “not at all,” “several days,” “more than half the days” and “nearly every day,” respectively. Higher scores mean more severe anxiety. It was scored for the severity of anxiety as follows: 0–4, none; 5–9, mild; 10–14, moderate; ≥15, severe. Zero was taken as negative while ≥1 was regarded as positive for each item of GAD-7. A total score ≥ 5 was considered positive for GAD-7 evaluation.

#### 2.2.4. PHQ-9

PHQ-9 is used to evaluate the signs and symptoms of depression within the last 2 weeks through items reflecting nine DSM-5 criteria of major depression (Kroenke et al., 2001). PHQ-9 is calculated by assigning scores of 0, 1, 2 and 3, corresponding to “not at all,” “several days,” “more than half the days,” and “nearly every day,” respectively. A total score (0–27) was obtained by adding together all items. It was scored for the severity of depression as follows: 0–4, none; 5–9, mild; 10–14, moderate; ≥15, severe. Zero was taken as negative while ≥1 was regarded as positive for each item of PHQ-9. A total score ≥ 5 was considered positive for the PHQ-9 evaluation. Moreover, if the score of the last item of PHQ-9 was ≥1, it was also considered positive even though the total score was <5.

### 2.3. Statistical analysis

Statistical analysis was performed on Statistical Package for Social Sciences version 22.0. Data drawing was completed by GraphPad Prism 8.0 software. Quantitative variables were expressed as mean ± standard deviation (M ± SD), and qualitative variables were expressed as numbers and percentages. Differences between two and multiple groups were analyzed by Student’s *t*-test and one-way ANOVA, respectively. Categorical variables were compared using chi-squared statistic tests. Taking the diagnostic results of PHQ-15 as the gold standard, the ability of SSS-CN to diagnose SSD was analyzed by the receiver operating characteristic (ROC) curve, and the area under the curve (AUC) value, sensitivity, and specificity were calculated. Reliability analysis was performed with the *Kappa* statistic to determine consistency between two scales of SSD. Two scales were considered to be in slight agreement if 0.00 to 0.20, fair agreement if 0.21 to 0.40, moderate agreement if 0.41 to 0.60, substantial agreement if 0.61 to 0.80, and in perfect agreement if it was 0.81 to 1.00. Coefficients of correlation (*r*) were analyzed by *Spearman* correlation analysis. All *p*-values were 2-tailed, and *p* < 0.05 was considered statistically significant.

## 3. Results

### 3.1. Demographics characteristics

1,102 patients were evaluated for SSD by SSS-CN. 1,152 patients were evaluated for SSD by PHQ-15 (*n* = 1,324). According to the scores of SSS-CN and PHQ-15, all patients were divided into three groups: mild, moderate, and severe SSD, respectively. There was no significant difference in occupation among the three groups evaluated by SSS-CN or PHQ-15. However, there were significant differences in the percentage of females, education level, and score of each scale among the three groups (all *p* < 0.05), whether patients were grouped according to SSS-CN or PHQ-15. The significant difference in age among the three groups was only found when patients were evaluated by SSS-CN (*p* < 0.05), i.e., those with severe SSD were significantly older than others (Table 1).

**Table 1:** Demographics characteristics of subjects with different SSD assessed by SSS-CN and PHQ-15.

	SSS-CN				PHQ-15			
	Mild (30–39)	Moderate (40–59)	Severe (≥ 60)	*p* value	Mild (5–9)	Moderate (10–14)	Severe (≥15)	*p* value
*n* (%)	485 (44.0)	531 (48.2)	86 (7.8)	-	440 (38.2)	454 (39.4)	258 (22.4)	-
Age, y	47.8 ± 15.8	49.8 ± 15.0	53.2 ± 12.8	0.005	47.6 ± 16.1	49.3 ± 15.1	49.9 ± 13.8	0.990
Female, *n* (%)	264 (54.4)	344 (64.8)	57 (66.3)	0.002	233 (53.0)	272 (59.9)	196 (76.0)	0.000
Education, *n* (%)				0.000				0.001
≤Primary	87 (17.9)	136 (25.6)	27 (31.4)		79 (18.0)	109 (24.0)	68 (26.4)	
Secondary	250 (51.5)	288 (54.2)	46 (53.5)		217 (49.3)	242 (53.3)	137 (53.1)	
≥College	148 (30.6)	107 (20.2)	13 (15.1)		144 (32.7)	103 (22.7)	53 (20.5)	
Occupation, *n* (%)				0.652				0.331
Working	371 (76.5)	397 (74.8)	61 (71.0)		334 (75.9)	345 (76.0)	187 (72.5)	
Student	14 (2.9)	20 (3.8)	2 (2.3)		19 (4.3)	16 (3.5)	6 (2.3)	
Retirement	100 (20.6)	114 (21.4)	23 (26.7)		87 (19.8)	93 (20.5)	65 (25.2)	
Score								
SSS-CN	34.6 ± 2.7	47.7 ± 5.5	65.1 ± 4.1	0.000	35.4 ± 7.4	43.5 ± 9.3	51.8 ± 9.8	0.000
PHQ-15	8.8 ± 3.6	12.8 ± 4.0	16.5 ± 4.4	0.000	7.2 ± 1.4	11.8 ± 1.3	17.6 ± 2.7	0.000

Data were expressed as mean ± standard deviation or *n* (%). SSD, somatization symptom disorders; SSS-CN, somatic symptom scale-China; PHQ-15, patient health questionnaire-15.

### 3.2. Detection rate and distribution of scale score of SSS-CN or PHQ-15

**Figure 2:**
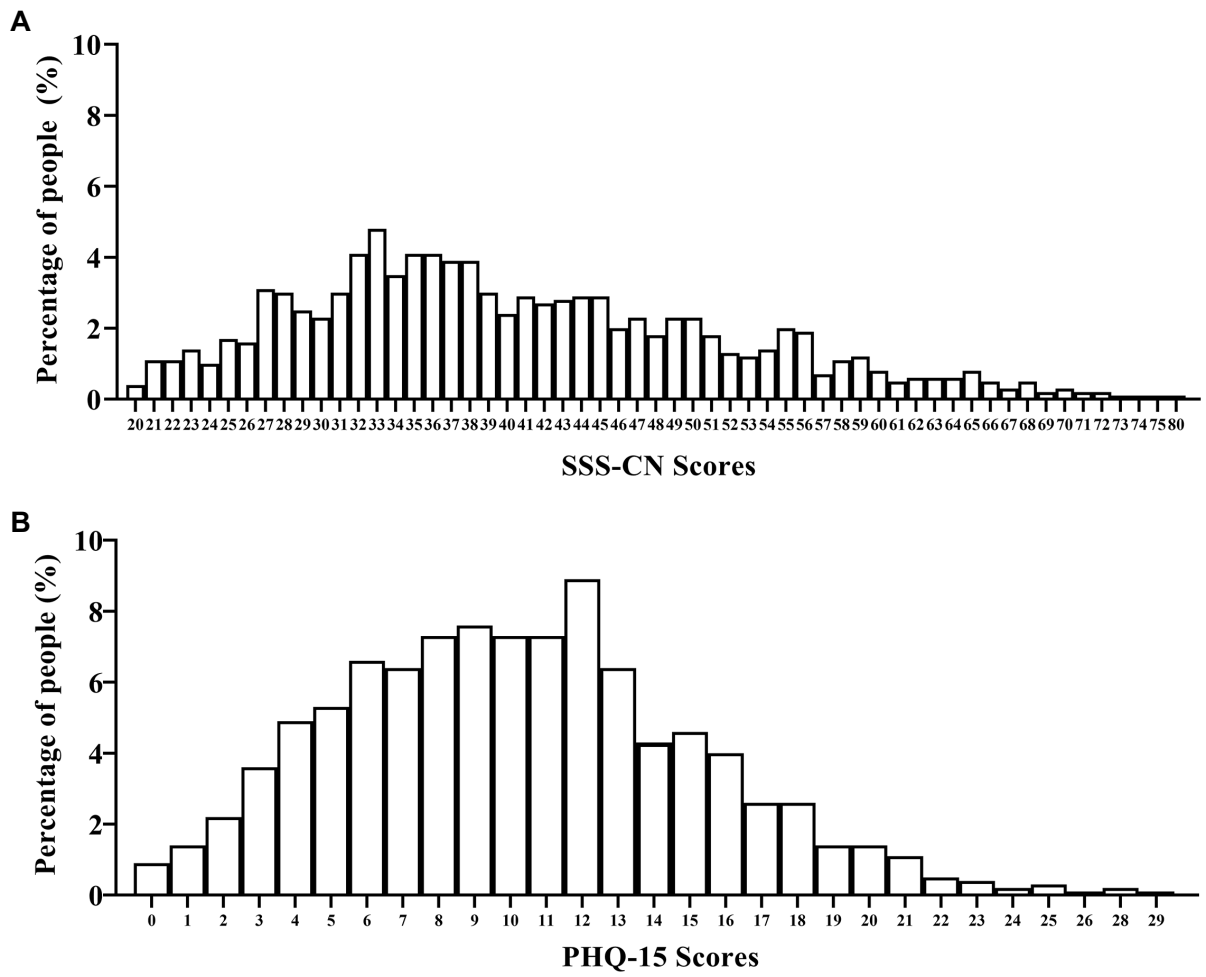
Scores distribution and the corresponding percentages in all subjects evaluated by SSS-CN or PHQ-15. **(A)** All subjects were evaluated by SSS-CN. **(B)** All subjects were evaluated by PHQ-15. *n* = 1,324.

When patients were evaluated by SSS-CN and PHQ-15, SSD was detected in 83.2 and 87.0%, respectively. No matter which scale was used, mild SSD was detected in more than 30% ones (SSS-CN: 36.7%, PHQ-15: 33.2%). The percentages of moderate SSD were highest (SSS-CN: 40.1%, PHQ-15: 34.3%), while those of severe SSD was lowest (SSS-CN: 6.5%, PHQ-15: 19.5%). Although the number of items differed between the SSS-CN and PHQ-15, the SSS-CN or PHQ-15 scores were similar and were positively skewed (Figure 2).

### 3.3. Positive rate of each item in SSS-CN or PHQ-15

**Figure 3:**
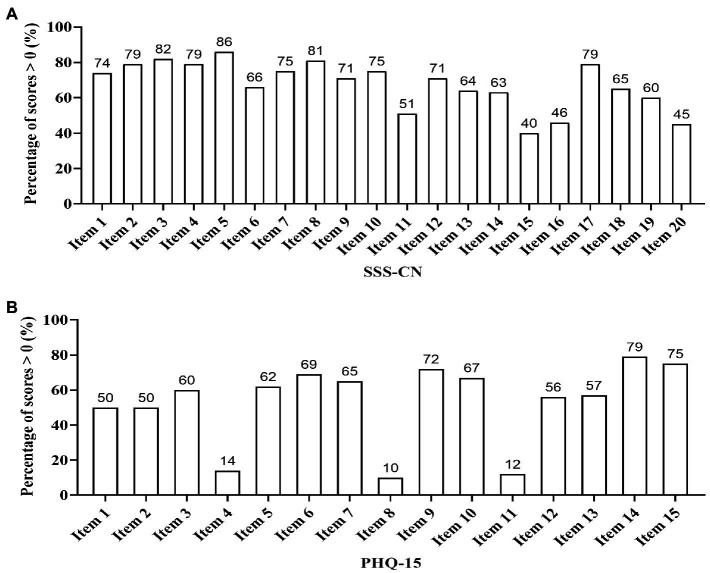
Positive rate of each item in SSS-CN or PHQ-15. **(A)** All subjects were evaluated by SSS-CN. Item 1, Dizziness, swelling in the head, heavy head, headache, spinning head, faint, buzzing in head. Item 2, Trouble sleeping. Item 3, Feeling tired or having low energy. Item 4, Losing interest, moody, do not want to be bothered, lacking patience. Item 5, Chest pain, shortness of breath, racing heart, chest tightness. Item 6, Easily anxious, nervous, feeling scared, panicky, feeling I’m going to die, out of control. Item 7, Worried, apprehensive, negative ideation. Item 8, Reduced attention and thinking abilities, forgetful, absentminded. Item 9, Bloating, stomach pain, gas, loss of appetite, constipation, loose bowels, nausea, becoming thin, dry or bitter mouth. Item 10, Pain in the neck, back, shoulders, waist, arm, legs. Item 11, Sensitive, easily sad and crying. Item 12, Unusual sensations in the joints of hands or legs. Item 13, Blurry vision, eye dryness, eye pain or swelling, decreased eye vision over a short period of time. Item 14, Easily agitated or irritable. Sensitive to voice, susceptible to startle. Item 15, Obsessive–compulsive thoughts or behaviors. Item 16, Skin allergies, itching, rash, skin flushing, hot flash, sweating. Item 17, Excess concerns about health issues, excessive worry that you or family members are ill. Item 18, Difficulty breathing, feeling oppressed or suffocated, frequent long sigh, coughing, intercostal pain. Item 19, Choking feeling in the throat, nasal dryness and obstruction, ringing in the ears or ear blockage. Item 20, Frequent urination, urgent need to urinate, painful urination, or discomfort in perineum. **(B)** All subjects were evaluated by PHQ-15. For the description of the PHQ-15, see reference (Kroenke et al., 2002).

The positive rate of each item in SSS-CN was at least or above 40%. The positive rates of the lowest three items (items 15, 16, and 20) were between 40 and 46%, and others were ≥51%. The positive rate of item 5, the question about “chest pain, shortness of breath, racing heart, chest tightness,” was the highest and reached 86%. The second highest one was item 3, the question about “feeling tired or having low energy,” and reached 82%. The third highest one was item 8, the question about “reduced attention and thinking abilities, forgetful, absentminded,” and reached 81%. Then followed by item 2, item 4, and item 17, the questions about “trouble sleeping,” “losing interest, moody, do not want to be bothered, lacking patience” and “excess concerns about health issues, excessive worry that you or family members are ill,” respectively, whose positive rate were all 79% (Figure 3A).

The positive rate of each item in PHQ-15 differed greatly. The positive rates of the lowest three items (items 4, 8, and 11) were between 10 and 14%, and others were ≥ 50%. The positive rate of item 14, the question about “feeling tired, or having low energy” was the highest and reached 79%. The second highest one was item 15, the question about “trouble sleeping,” and reached 75%. Then followed by item 9, item 6, and item 10, all of which were around 70%, corresponding to the question “feeling your heart pound or race,” “chest pain,” and “shortness of breath,” respectively. Conversely, the lowest one was item 8, the question about “fainting spells,” which was only 10%. Then followed by item 11 and item 4, corresponding to “pain or problems during sexual intercourse,” and “menstrual cramps or other problems with your periods,” which were 12 and 14%, respectively (Figure 3B).

### 3.4. Correlation between SSS-CN and PHQ-15

**Figure 4:**
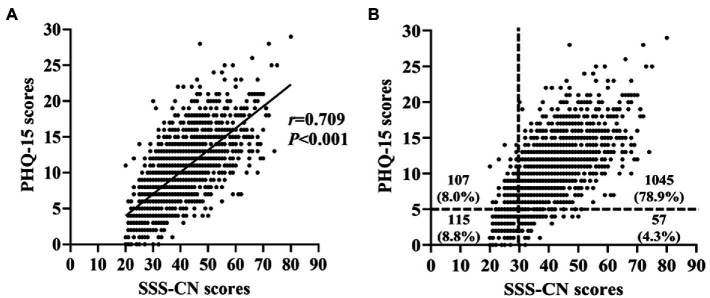
Correlation analysis between the SSS-CN scores and PHQ-15 scores. **(A)** Correlation between the scores of SSS-CN and those of PHQ-15. **(B)** Distribution of the SSS-CN and PHQ-15 scores in all subjects.

Correlation analysis showed that the SSS-CN score was highly and significantly correlated with PHQ-15 one (*r* = 0.709, *p* < 0.001, n = 1,324; Figure 4A). The number of patients who were double-positive when evaluated by both SSS-CN and PHQ-15 was 1,045 (78.9%). Analogously, the number of patients who were double negative when evaluated by both SSS-CN and PHQ-15 was 115 (8.8%). 57 patients were positive as assessed by SSS-CN and negative by PHQ-15, accounting for 4.3% of the total subjects. There were 107 patients assessed as negative by SSS-CN and positive by PHQ-15, accounting for 8.0% of the total subjects (Figure 4B).

### 3.5. Analysis of the diagnostic effect of SSS-CN on SSD

**Figure 5:**
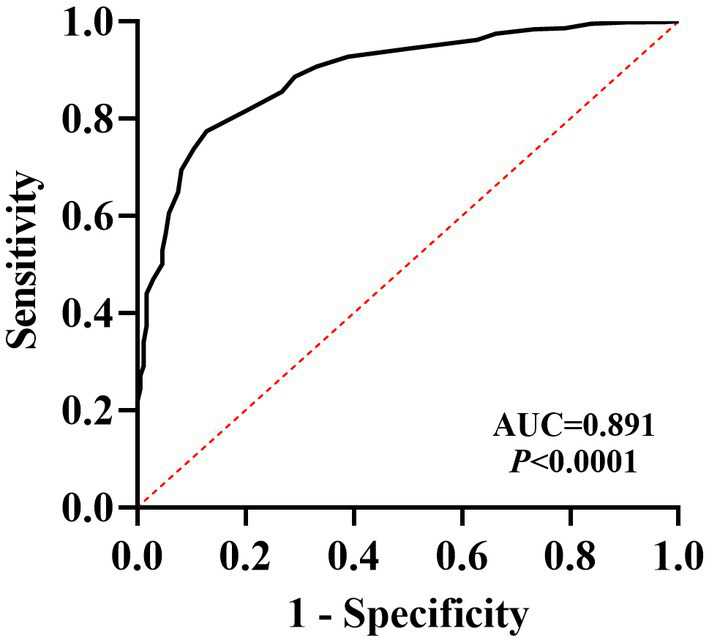
ROC curve analysis of SSS-CN in diagnosing SSD.

ROC curve analysis showed that the sensitivity of SSS-CN ranged from 87 to 90% and the specificity ranged from 64 to 77% when a cut-off value of 30 was taken. Area under the ROC curve = 0.891 (Figure 5). Then, *Kappa* analysis was performed to assess the consistency between SSS-CN and PHQ-15 in the diagnosis of SSD. The value for *Kappa* analysis was 0.512, indicating a moderate agreement between SSS-CN and PHQ-15 in evaluating SSD.

### 3.6. Association of SSD with anxiety and depression

Of all suspected subjects (n = 1,324), 687 (51.9%) were assessed positive for GAD-7, 702 (53.0%) were assessed positive for PHQ-9, and 547 (41.3.0%) were assessed positive for both GAD-7 and PHQ-9. A total of 842 (63.6%) were detected as having an emotional disorder, i.e., anxiety or/and depression.

There were 1,102 subjects with SSD assessed by the SSS-CN, of whom 652 were positive for GAD-7, 677 were positive for PHQ-9, and 534 were positive for both GAD-7 and PHQ-9. There were 222 subjects assessed as negative by SSS-CN. Among them, 35 cases were positive for GAD-7, 25 cases were positive for PHQ-9, and 13 cases were positive for both GAD-7 and PHQ-9. There were 1,152 patients with SSD assessed by PHQ-15, 648 cases were positive for PHQ-9, 677 cases were positive for PHQ-9, and 530 cases were positive for both GAD-7 and PHQ-9. There were 172 negative subjects assessed by PHQ-15, among them, 39 were positive for GAD-7, 25 were positive for PHQ-9, and 16 were positive for both GAD-7 and PHQ-9. In total, 795 ones with anxiety or/and depression were detected in patients with SSD evaluated by either SSS-CN (72.1%) or PHQ-15 (69.0%).

**Figure 6:**
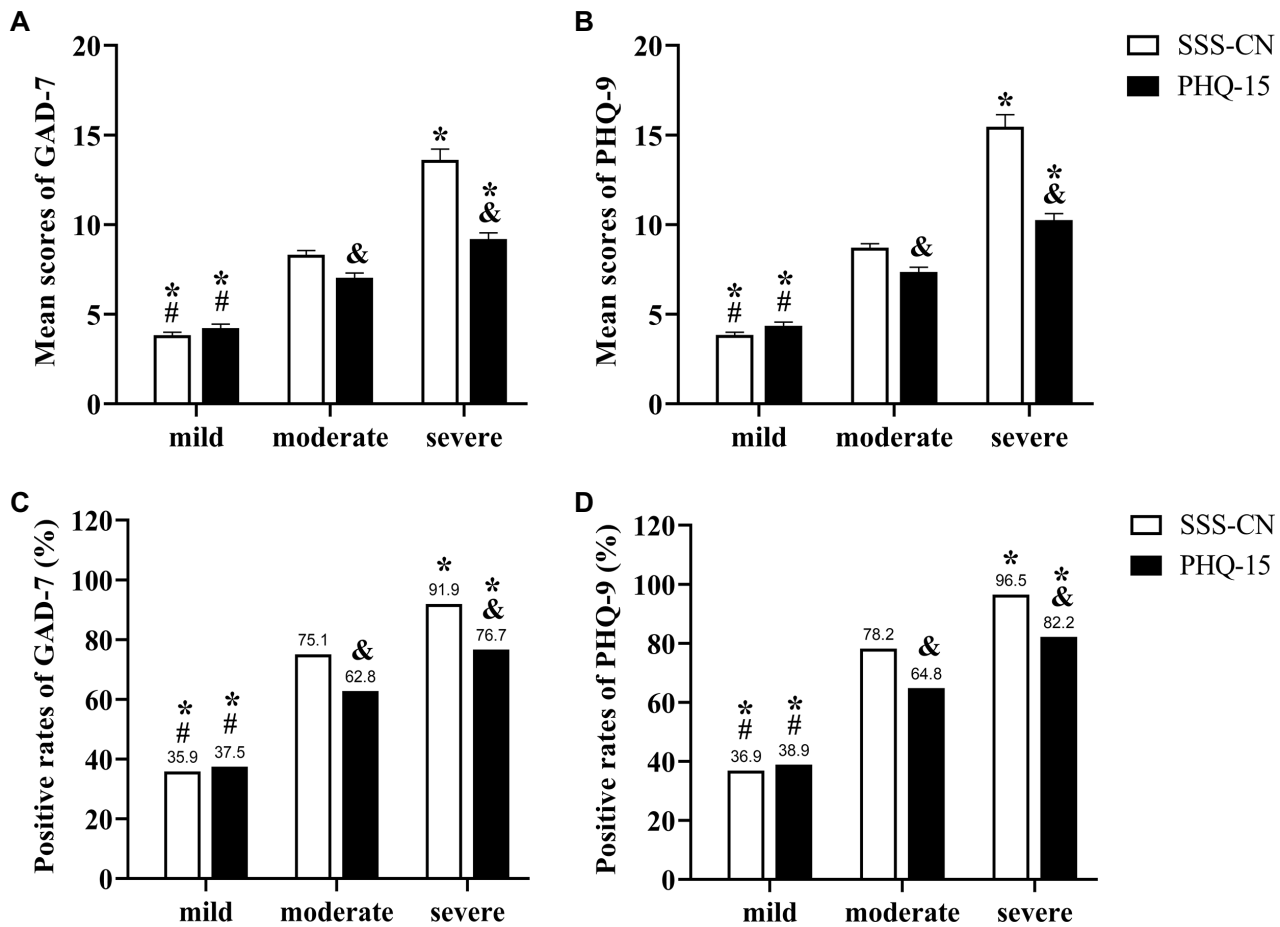
Comparison of GAD-7 or PHQ-9 scores and positive rates in patients with different degrees of SSD. **(A,B)** The mean scores of GAD-7 **(A)** or PHQ-9 **(B)** in subjects with different degrees of SSD were evaluated by SSS-CN or PHQ-15. **(C,D)** The positive rates of GAD-7 **(C)** or PHQ-9 **(D)** in subjects with different degrees of SSD evaluated by SSS-CN or PHQ-15. *p < 0.05 When compared with the subjects with moderate SSD. ^#^*p* < 0.05 When compared with the subjects with severe SSD. ^&^*p* < 0.05 When compared with the subjects within the same degrees of SSD evaluated by SSS-CN.

Whether SSD was assessed with the SSS-CN or PHQ-15, the GAD-7 or PHQ-9 scores and positive rates gradually increased in patients with mild, moderate, and severe SSD, with significant differences among the three groups (*p* < 0.05), suggesting that the more severe the SSD, the higher the GAD-7 or PHQ-9 scores and positive rates (Figure 6).

Notably, patients with moderate and severe SSD evaluated by SSS-CN had a significantly higher score and positive rate of GAD-7 or PHQ-9 than those evaluated by PHQ-15 (*p* < 0.05). Among patients with moderate SSD assessed by PHQ-15, the positive rates of anxiety and depression were 62.8 and 64.8%, respectively. Among patients with moderate SSD assessed by the SSS-CN, the positive rates of anxiety and depression were 75.1 and 78.2%, respectively. In patients with severe SSD assessed by PHQ-15, the positive rates of anxiety and depression were 76.7 and 82.2%, respectively. Among patients with severe SSD assessed by SSS-CN, the positive rates of anxiety and depression were as high as 91.9 and 96.5%, respectively (Figure 6).

**Figure 7:**
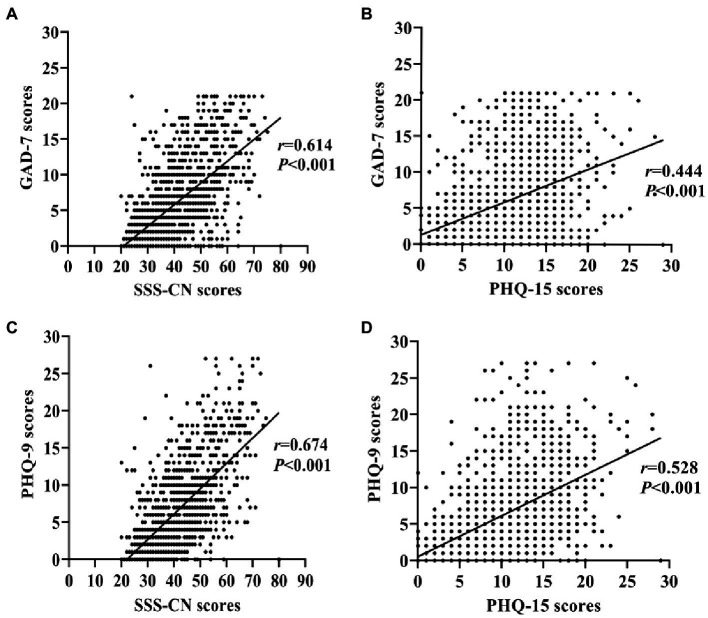
Correlation analysis between the scores of SSS-CN or PHQ-15 with those of GAD-7 or PHQ-9 in the total subjects. **(A)** Correlation between the scores of SSS-CN and GAD-7. **(B)** Correlation between the scores of PHQ-15 and GAD-7. **(C)** Correlation between the scores of SSS-CN and PHQ-9. **(D)** Correlation between the scores of PHQ-15 and PHQ-9. Spearman correlation analysis was performed.

Correlation analysis showed that SSS-CN score was highly and significantly correlated with GAD-7 (*r* = 0.614, *p* < 0.001) or PHQ-9 one (*r* = 0.674, *p* < 0.001; Figures 7A,C). PHQ-15 score was also significantly correlated with GAD-7 (*r* = 0.444, *p* < 0.001) and PHQ-9 one (*r* = 0.582, *p* < 0.001), respectively (Figures 7B,D). It seemed that the SSS-CN score had a stronger association with GAD-7 or PHQ-9 one than the PHQ-15 score.

## 4. Discussion

The present study is the first one to compare the clinical application value of SSS-CN with that of PHQ-15 in more than 1,000 Chinese patients with suspected psychological disorders screened by a “three-question method” in the department of cardiovascular medicine. It was shown that the detection rate of SSD was above 80% by two self-rating scales. The score of SSS-CN was highly correlated with that of PHQ-15. The area under the ROC curve = 0.891, reflecting that the SSS-CN has good accuracy. More importantly, the SSS-CN score was more closely correlated with the GAD-7 or PHQ-9 score than the PHQ-15 score, and higher proportion of patients with anxiety or depression was detected in those with moderate and severe SSD evaluated by SSS-CN. It indicated that the SSS-CN can not only replace the PHQ-15 in assessing Chinese patients with SSD but also correlate better with anxiety or/and depression than the PHQ-15, especially in patients with moderate/severe SSD assessed by the SSS-CN.

Zhang et al. (2019) previously reported that the detection rate of SSD evaluated by PHQ-15 was 93.1% in 508 cardiovascular outpatients with suspected psychological disorders screened by a “three-question method.” When 1,324 cardiovascular patients with suspected psychological disorders were evaluated by PHQ-15 in this study, the detection rate of SSD was 87.0% which was slightly lower than the rate of Zhang et al. (2019). However, in the primary care patients of Qatar and Germany, the detection rate of SSD evaluated by PHQ-15 was only 11.7 and 18.4%, respectively (Hanel et al., 2009; Bener et al., 2013). The difference in the detection rate of SSD among those studies indicated that a “three-question method” was really helpful to screen out patients with SSD and elevate the detection rate of SSD, at least in Chinese patients.

In this study, although the PHQ-15 detected more patients with SSD than the SSS-CN (1,152: 1102), the ROC curve analysis showed that the AUC of the SSS-CN for the diagnosis of SSD was 0.891, indicating that the scale has good accuracy and high sensitivity of more than 90%, with a specificity within an acceptable range. We noted that the top 5 items with a high positive rate in PHQ-15 were similar to those in SSS-CN. In addition to item 5 inquiring about somatic disorders, other items (items 2, 3, 4, 8, and 17) within the top 6 ones of SSS-CN in this study inquired about emotional disorders, including anxiety, depression, anxiety, and depression. This is also a prominent difference between SSS-CN and PHQ-15. The latter only investigates somatic symptoms and does not involve emotional disorders.

Interestingly, there were 3 items with a low positive rate of less than 15% when the suspected patients were evaluated by PHQ-15 in this study. For example, the positive rate of item 11 inquiring about pain or problems during the sexual intercourse was only 12%. Similarly, Zhang et al. (2019) reported a lower positive rate of item 11, only 4.65%, within the suspected outpatients in the department of cardiovascular medicine. The other two items with low positive rates were item 4 inquiring about menstrual cramps and item 8 inquiring about fainting spells. Low positive rates of reproduction-related items could be related to nearly half female and relatively older participants in this study. More than that, the Chinese were more conservative than westerners, and often rejected sex-related issues due to a variety of factors, such as stigma, and so on. Even if they had serious sexual problems, they often ignored them or hid them from doctors, which may be related to cultural differences between the East and the West.

Psychological disorders can manifest as physical discomforts. It was shown that more than half of the patients with depression and/or anxiety who visited the general hospitals only complained about physical symptoms (Kroenke, 2003; Devane et al., 2005). On the other hand, patients with cardiovascular diseases could also be complicated with emotional disorders, i.e., anxiety and/or depression. In this study, emotional disorders were detected in about 70% of patients with SSD evaluated by either SSS-CN or PHQ-15. Between two groups of patients with SSD evaluated by SSS-CN and PHQ-15, there was barely a difference in the absolute numbers of patients with anxiety, depression, and anxiety combining depression although SSS-CN-evaluated patients were less than PHQ-15-evaluated patients (1,102: 1152). Moreover, the absolute numbers of SSD patients with anxiety or/and depression were the same between the two groups, accounting for 94.4% of all patients with anxiety or/and depression (795 of 842) in this study. Moreover, the proportion of patients with SSD combined with emotional disorders (70%) was higher than those reported in two previous studies completed in Qatar and German primary care clinics (60.67 and 46.74%, respectively; Hanel et al., 2009; Bener et al., 2013). This suggests that a “three-question method” of primary screening not only helps to increase the detection rate of SSD but also helps to screen patients with comorbidity emotional disorders.

In this study, the SSS-CN score was more closely related to GAD-7 or PHQ-9 than the PHQ-15 score, although there was a positive correlation between the score of each SSD scale and that of the anxiety or depression scale. More importantly, patients with moderate-to-severe SSD assessed by the SSS-CN had higher scores on the GAD-7 or PHQ-9 and higher rates of anxiety or depression. This may be due to that the SSS-CN contains items involving emotional disorders, such as those involving anxiety alone (items 6, 14, 15, and 17), depression alone (items 3, 4, 7, and 11), or both anxiety and depression (items 2 and 8; Jiang et al., 2019). Because SSS-CN covers symptoms of both somatic and emotional disorders, it could be efficient to predict more patients with anxiety or depression when SSS-CN-evaluated SSD was moderate and severe. This feature of SSS-CN is really practical. When SSD coexists with anxiety or/and depression, especially when Eastern patients are more willing to complain of physical discomfort than to mention psychological abnormalities (Ryder et al., 2008), this poses greater difficulty for clinicians to diagnose psychological disorders and makes their diagnosis less accurate (Axelsson et al., 2016). In the busier outpatient departments in China, cardiovascular physicians can use the SSS-CN to make initial judgments about patients with suspected psychological disorders and focus on detecting patients with moderate to severe SSD and referring them quickly to the corresponding specialty if necessary.

We need to acknowledge that in addition to the SSS-CN and PHQ-15 scales, there are a number of other scales used to assess SSD, such as the Symptom Check List-90 (SCL-90) and the Somatic Symptom Scale-8 (SSS-8). The SCL-90 is a traditional symptom self-rating scale consisting of 90 entries (Sereda and Dembitskyi, 2016), whereas the SSS-8, a short version of the PHQ-15, was developed for application in settings where measurement time is limited (Gierk et al., 2014). Both self-assessment scales have been shown to have good measurement properties (Gierk et al., 2014; Sereda and Dembitskyi, 2016). In similar studies conducted in the future, we will consider the addition of these scales to ensure the reliability and validity of the SSS-CN.

There were several limitations in this study. First, there was a lack of psychiatrists to evaluate the suspected patients. The setting of a combined cardiac-psychological clinic could better solve this problem. Second, only patients in the department of cardiovascular medicine were explored. It is worth to exploring the application of SSS-CN in patients in other comprehensive departments in the future. Third, this study was a cross-sectional one. The change in SSS-CN score after treatment was not assessed.

## 5. Conclusion

There was moderate agreement between the SSS-CN and the PHQ-15 in assessing SSD in patients with suspected psychological disorders in cardiovascular medicine. Both SSD self-assessment scale scores were highly positively correlated with anxiety or depression self-assessment scale scores. SSS-CN scores were more strongly correlated with GAD-7 or PHQ-9 scores than PHQ-15 scores. The SSS-CN could be one of the ideal scales for the rapid screening of patients with suspected psychological disorders in cardiovascular medicine.

## Data availability statement

The original contributions presented in the study are included in the article/supplementary material, further inquiries can be directed to the corresponding author/s.

## Ethics statement

The observational research was discussed and approved by the Ethics Committee of the Second Xiangya Hospital of Central South University. All procedures performed in this study involving human participants were in accordance with the ethical standards of the institutional and/or national research committee and with the 1964 Helsinki declaration and its later amendments or comparable ethical standards. The patients/participants provided their written informed consent to participate in this study.

## Author contributions

QL, QX, and LL designed and conducted of this study. YF, QL, and QX participated in the collection of the data. YF and QL analyzed the data and interpreted the results. YF wrote the paper. LL and XW conducted the literature review. All authors contributed to the article and approved the submitted version.

## Funding

This project was supported by the National Natural Science Foundation of China (Grant Numbers: 81270956 and 81470577) and the Funds of Central South University (Grant number: XCX2021305).

## Conflict of interest

The authors declare that the research was conducted in the absence of any commercial or financial relationships that could be construed as a potential conflict of interest.

## Publisher’s note

All claims expressed in this article are solely those of the authors and do not necessarily represent those of their affiliated organizations, or those of the publisher, the editors and the reviewers. Any product that may be evaluated in this article, or claim that may be made by its manufacturer, is not guaranteed or endorsed by the publisher.

## Abbreviations

SSD: Somatization symptom disorders
PHQ-15: Patient health questionnaire-15
SSS-CN: Somatic symptom scale-China
PHQ-9: Patient health questionnaire-9
GAD-7: General anxiety disorder-7
SCL-90: Symptom check list-90
SSS-8: Somatic symptom scale-8
ROC: The receiver operating characteristic
AUC: The area under the curve
*r*: Coefficients of correlation
